# Loudness dependence of auditory evoked potentials reflects trait anxiety and harm avoidance in healthy adults: an exploratory study

**DOI:** 10.3389/fnhum.2025.1615407

**Published:** 2025-10-15

**Authors:** Kohei Fujita, Nobuyuki Takeuchi, Shunsuke Sugiyama, Koji Inui, Yuki Fujita, Ami Yamaba, Taeko Kamiya, Takahiro Ushida, Kousuke Kanemoto, Jun Miyata, Makoto Nishihara

**Affiliations:** ^1^Neuropsychiatric Department, Aichi Medical University, Nagakute, Japan; ^2^Bijutsukanmae Mental Clinic, Okazaki, Japan; ^3^Department of Psychiatry and Psychotherapy, Gifu University, Gifu, Japan; ^4^Department of Integrative Physiology, National Institute for Physiological Sciences, Okazaki, Japan; ^5^Department of Functioning and Disability, Institute for Developmental Research, Aichi Developmental Disability Center, Kasugai, Japan; ^6^Central Clinical Laboratory, Aichi Medical University Hospital, Nagakute, Japan; ^7^Multidisciplinary Pain Center, Aichi Medical University, Nagakute, Japan

**Keywords:** Loudness Dependence of Auditory Evoked Potentials (LDAEP), State-Trait Anxiety Inventory (STAI), Temperament and Character Inventory (TCI), anxiety, harm avoidance, novelty seeking, serotonin

## Abstract

Loudness dependence of auditory-evoked potentials (LDAEP), a neurophysiological measure that reflects central serotonergic activity, is also influenced by the noradrenaline and dopamine systems. While it has been used in investigations of various psychiatric disorders, the fundamental characteristics in healthy individuals remain largely unknown. The present exploratory study examined 60 healthy adults to determine the associations of LDAEP with psychological traits assessed using the Temperament and Character Inventory (TCI) and State-Trait Anxiety Inventory (STAI). The participants completed TCI and STAI questionnaires before undergoing electrophysiological recordings. LDAEP was measured based on the peak-to-peak amplitude slopes of P50/N100 and N100/P200 across five sound intensity levels. Using Spearman's correlation analysis, moderate negative correlations of LDAEP slopes with harm avoidance (HA) and trait anxiety (STAI-T) scores were noted. Additionally, sex-adjusted analysis showed novelty seeking to be positively associated with LDAEP. These findings suggest an association of LDAEP with individual differences in harm avoidance, trait anxiety, and novelty seeking in healthy adults. This supports the potential use of LDAEP as a non-invasive biomarker to predict treatment response in major depressive disorder, as well as in anxiety-related conditions.

## 1 Introduction

Loudness dependence of auditory-evoked potentials (LDAEP) is a neurophysiological measure used to assess cortical responses to various intensities of auditory stimuli. It is measured using sound pressures of approximately 4–6 different sound pressure levels and quantified by calculating the slopes of the increase in amplitude of evoked potentials (Hegerl and Juckel, 1993). Although the method for determining LDAEP has not been fully standardized, a protocol that uses five auditory stimuli and recording from the Cz electrode is widely employed (Park et al., 2011). Although early animal studies suggested that a low LDAEP reflects high serotonergic activity (Juckel et al., 1997; Wutzler et al., 2008), more recent findings indicate that LDAEP is also influenced by noradrenergic and dopaminergic systems, and thus is not solely regulated by serotonergic activity (Lee et al., 2011; Fitzgerald, 2024).

Previous studies have examined the association of LDAEP with various psychiatric disorders, such as major depressive disorder (MDD) (Leuchter et al., 2009), generalized anxiety disorder (GAD) (Park et al., 2011), bipolar disorder (Lee et al., 2012; Hagenmuller et al., 2016), and schizophrenia (Juckel, 2015). However, results of studies on the role of LDAEP as a diagnostic biomarker are contradictory (Roser et al., 2020). On the other hand, LDAEP has been shown to have significant potential as a biomarker for predicting treatment responses, with meta-analysis and clinical studies validating its role in predicting responses to selective serotonin reuptake inhibitors (SSRIs) in individuals with MDD or GAD, with higher LDAEP generally associated with more favorable responses and lower LDAEP with poorer responses (Yoon et al., 2021). Based on these characteristics, LDAEP has been proposed as a potential biomarker for use in precision psychiatry, though additional findings for validation are required.

While the clinical utility of LDAEP has been suggested, it is considered important to investigate its fundamental characteristics in healthy adults. Notably, relationships between LDAEP and certain psychological indicators, such as novelty seeking (Juckel et al., 1995), impulsivity (Kim et al., 2016), and optimism (Zhang et al., 2013), have been reported, though comprehensive understanding has yet to be achieved. The present study was thus conducted as an exploratory investigation to identify potential associations between LDAEP and various psychological traits.

The relationship between serotonin and anxiety has been well studied (Lin et al., 2023). For the present exploratory investigation, we speculated that anxiety may also be associated with LDAEP, which is considered to reflect central serotonergic function. For the analyses, the State-Trait Anxiety Inventory (STAI), a widely used self-report tool that separately assesses trait and state aspects of anxiety (Spielberger et al., 1970), was included, as well as harm avoidance (HA), a temperament trait that is part of the Temperament and Character Inventory (TCI) and shown to be closely associated with serotonergic function (Hansenne and Ansseau, 1999). The TCI, originally developed by Cloninger (1994), is a psychological assessment tool used for determining associations of neurotransmitter systems (Munafò et al., 2005; Schosser et al., 2010). It was considered that analysis of the association between LDAEP and TCI would help elucidate the fundamental characteristics of LDAEP.

## 2 Methods

### 2.1 Participants

This study was performed in accordance with the Declaration of Helsinki and approved in advance by the Ethics Committee of the National Institute for Physiological Sciences, Okazaki, Japan. Participants were voluntarily recruited by use of advertisements posted within the hospital. Each provided written informed consent before participating in the study and anonymity was preserved. An oral confirmation meeting with each was then conducted by a psychiatrist and they were screened for a history of psychiatric, neurological, and/or substance use disorders. Sixty healthy volunteers (30 females, 30 males; mean age 35.5 years) with normal hearing based on self-reported data, no history of mental or neurological disorders or substance abuse within the last 5 years, and who did not use any medication during testing were subsequently enrolled. Sex was defined as biological sex assigned at birth, and categorized as either male or female. As this study involved healthy participants and focused on observational correlations, it was not classified as a clinical trial.

### 2.2 Auditory stimuli

For the LDAEP, an 80 ms pure tone at 800 Hz (rise/fall 10 ms to prevent undesired edges) was presented at five different sound pressure levels (55, 65, 75, 85, 95 dB SPL). For each sound category, 100 to 120 stimuli were presented. Inter-stimulus intervals were randomized between 1,800 and 2,200 ms. The tones of the five intensities were intermixed and presented randomly without restriction. All auditory stimuli were created using a personal computer (Windows XP 32-bit) and presented binaurally via earpieces (E-A-Rtone 3A; Aero Company, Indianapolis, IN, USA), with the level calibrated for each experiment using a sound level meter (Rion NL-32).

### 2.3 Electroencephalogram recordings and analysis

Each participant sat on a comfortable chair in a quiet, electrically shielded room and watched a silent movie. There were asked to ignore any sound stimuli. An exploring electrode was placed at the midline of the central site (Cz), referred to as the linked mastoid (single-electrode method), and a pair of electrodes was placed on the supra- and infra-orbits of the left eye, which were used to record the electrooculogram. The electroencephalogram artifact rejection level was set at 100 μV. When simultaneously recorded electrooculogram signals were >100 μV, the epoch was removed. The impedance of all electrodes was maintained at < 5 kΩ. AEPs were recorded using a band-pass filter ranging from 0.1–100 Hz (Neuropack MEB-2300; Nihon Kohden, Tokyo) at a sampling rate of 1,000 Hz. Before sound onset, the baseline was set at 100 ms, with an average of at least 100 epochs for LDAEP obtained.

Following each epoch, the AEP components were analyzed, with a 0.98–35.2 Hz digital filter applied to the continuous data offline at the zero phase, 24 dB/octave. The sound onset evoked a triphasic response with peaks of approximately 50(P50), 100(N100), and 200(P200) ms, with peak amplitudes measured in the time windows of 30–80, 80–150, and 150–280 ms, respectively. Peak-to-peak amplitudes were calculated for P50/N100 and N100/P200 (Fujita et al., 2022). This procedure has been shown to decrease issues related to baseline shifts (Inui et al., 2010). LDAEP is generally analyzed as the slope of the amplitude/stimulus intensity function among five sound pressure levels. For the present study, the slope was calculated as a linear regression line of the amplitude of the five points (linear slope) (Herrmann et al., 2002) and expressed as amplitude change per 10 dB stimulus intensity difference (μV/10 dB).

### 2.4 Psychological indicators

Previous studies have established the reliability and validity of the STAI (Nakazato, 1982) and 125-item version of the TCI (Kijima, 1996) used in Japan. Prior to the LDAEP recording, each participant completed a psychological indicator assessment based on those tools. The STAI is a 40-item self-administered instrument that includes a two-item scale (Spielberger et al., 1970). For the present participants, the State-Trait Anxiety Inventory-State Anxiety version (STAI-S) was used to assess current anxiety and the State-Trait Anxiety Inventory-Trait Anxiety version (STAI-T) to examine anxiety proneness. TCI comprises four temperaments, novelty seeking (NS), reward dependence (RD), HA, and persistence (P), as well as three personality traits, self-directedness (SD), cooperativeness (C), and self-transcendence (ST) (Cloninger, 1994).

### 2.5 Statistical analysis

A Shapiro–Wilk test was initially applied to assess normality of the obtained data, which revealed that NS, HA, P, SD, C, and ST were not normally distributed. Spearman's rank correlation analysis was then performed to determine associations among all variables. Statistical significance was set at p < 0.05. In addition to reporting uncorrected *p* values, false discovery rate (FDR) correction for multiple comparisons was applied using the Benjamini-Hochberg procedure to the set of pairwise correlation analyses among LDAEP slopes, STAI scores, TCI scores, and age. Uncorrected and FDR-corrected *p* values, as well as correlation coefficients, are presented in Supplementary Table 1. The analyses were conducted to generate hypotheses regarding potential associations between LDAEP and psychological traits. Furthermore, sub-analysis was conducted using a *t*-test to examine possible sex differences for each variable. For variables with significant sex differences, partial correlation analysis was performed after adjusting for sex. The absolute value of the correlation coefficients indicated a weak correlation at 0.1 < *r* < 0.3, moderate at 0.3 ≤ *r* < 0.5, and strong at *r* ≥ 0.5 (Cohen, 2013). All statistical analyses were conducted using IBM SPSS Statistics for Windows, version 25.0 (IBM Corp., Armonk, NY), with Microsoft Excel 2019 (Microsoft Corporation, Redmond, WA, USA) used for data entry and FDR correction. The datasets analyzed in the current study are available from the corresponding author upon request.

## 3 Results

**Figure 1:**
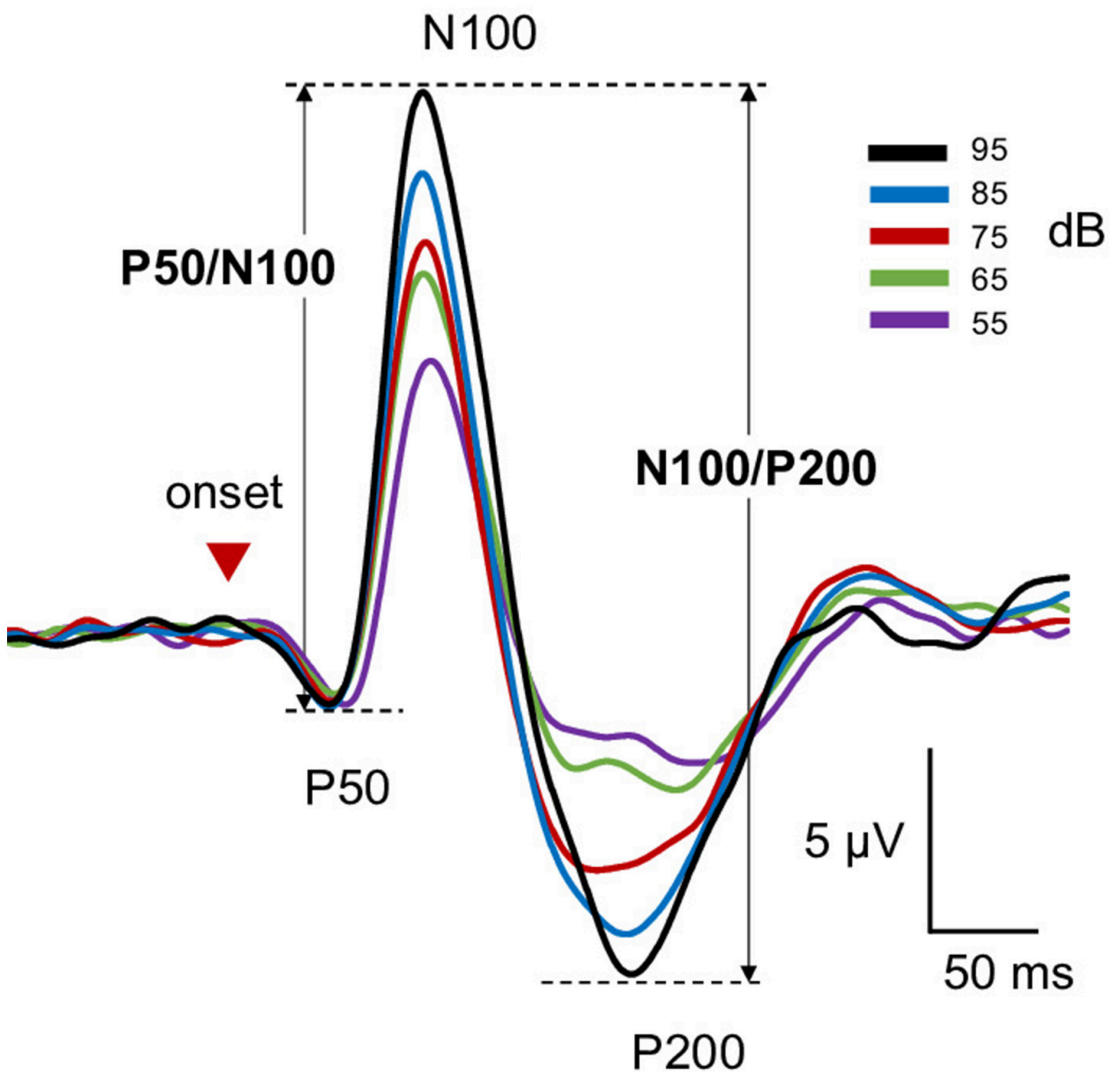
Grand-averaged wave forms of LDAEP responses. Shown are grand-averaged waveforms of LDAEP responses for each condition. Red arrowheads indicate sound onset.

**Figure 2:**
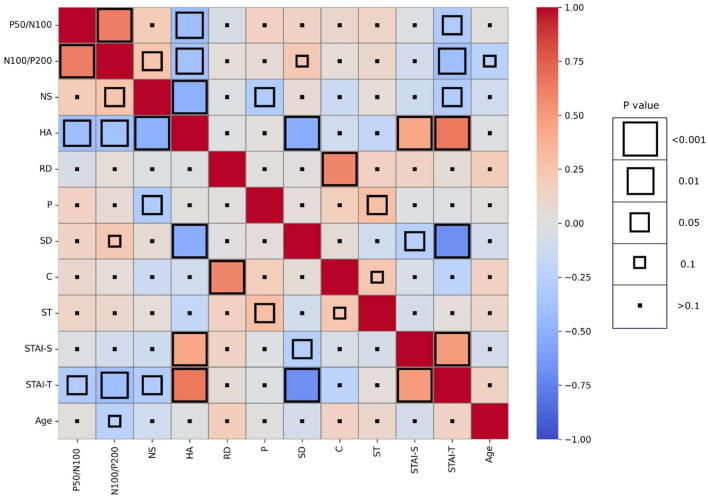
Heatmap showing pairwise Spearman correlation coefficients between LDAEP, psychological measures, and age (uncorrected *p*-values). Color intensity indicates the strength and direction of the correlation, while the size of each square represents the statistical significance.

**Figure 3:**
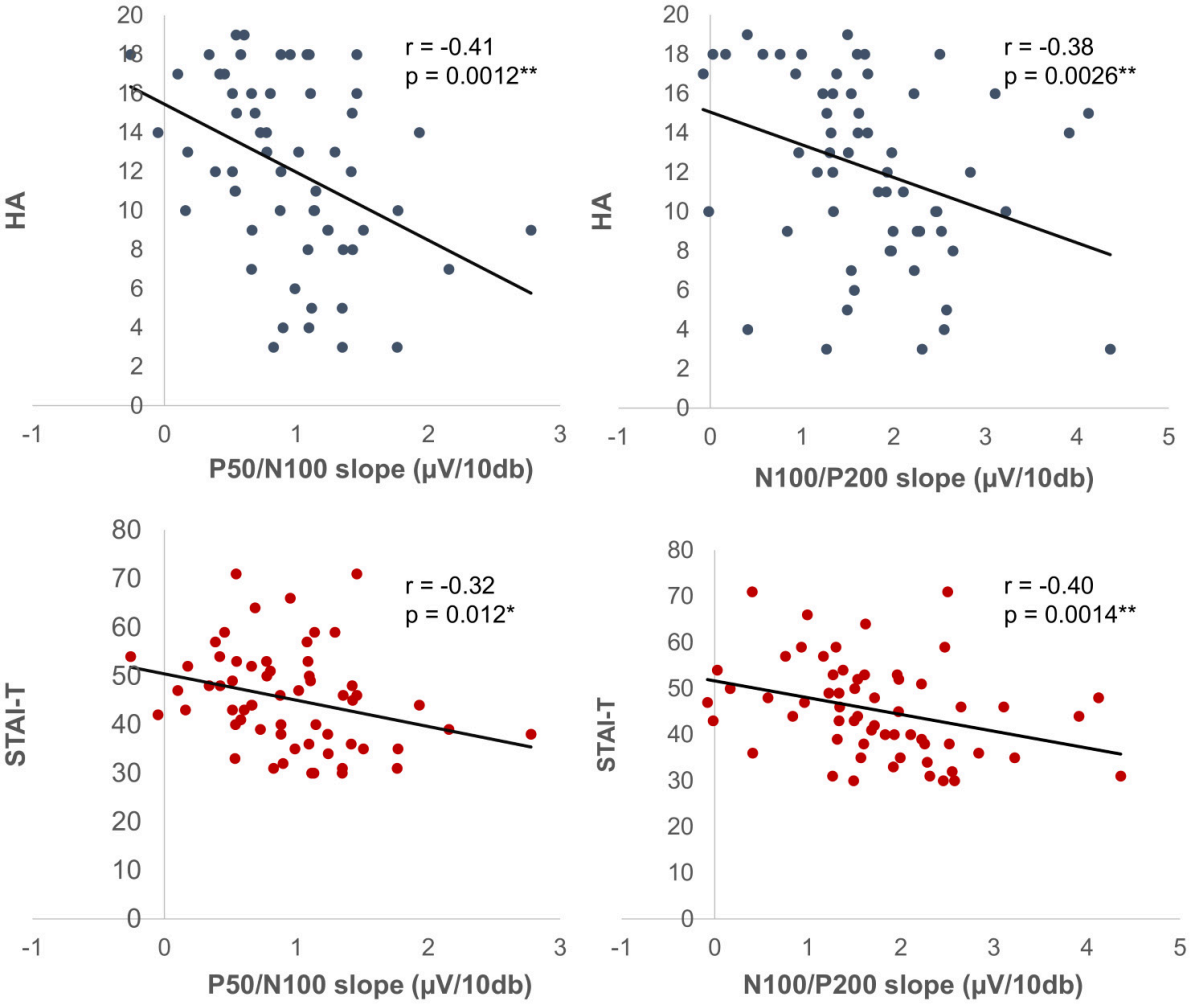
Correlations among amplitude slope, LDAEP, and psychological indicators. Shown are correlations among LDAEP, TCI-HA, STAI-T, P50/N100, and N100/P200 prior to FDR correction. ^*^P < 0.05. ^**^P < 0.01.

Mean amplitude, latency, and slope of the participants are presented in Table 1, while grand-averaged waveforms are shown in Figure 1. Age was not significantly different between females and males (*t*-test, *p* = 0.32). Sex showed no significant effect on LDAEP, STAI, or TCI scores (*t*-test, *p* > 0.1), while NS was significantly different between females and males (*p* = 0.0090) (Table 2). Therefore, other than NS, the analyses were not corrected for age or sex. As for anxiety score, 28 participants (46.7%) scored ≥40 on the STAI-S and 41 (68.3%) scored ≥40 on the STAI-T, which are thresholds commonly used to indicate a moderate or higher level of anxiety (Spielberger et al., 1970). To assess the reliability of the psychometric instruments, Cronbach's alpha coefficients were calculated. The internal consistency for each TCI scale ranged from acceptable to good (NS = 0.65, HA = 0.84, RD = 0.61, P = 0.57, SD = 0.82, C = 0.64, ST = 0.80), with the values found to be comparable to those reported in previous validation studies of the Japanese 125-item version of the TCI, such as that presented by Kijima (1996). Cronbach's alpha coefficients for the STAI scales were excellent (STAI-S = 0.91, STAI-T = 0.91), consistent with those reported in previous validation studies of the Japanese version (Nakazato, 1982) and considered to indicate a high level of internal consistency. Correlations between the LDAEP slopes, each of the STAI and TCI items, and age are presented in Figure 2. Notably, moderately significant correlations were observed between the P50/N100 slope and both STAI-T score (r = −0.32) and HA (r = −0.41), as well as between the N100/P200 slope and both STAI-T score (r = −0.40) and HA (r = −0.38) (*p* < 0.05 for each). To address the issue of multiple comparisons, FDR-corrected *p* values were also calculated using the Benjamini-Hochberg procedure (Supplementary Figure 1), with both raw and FDR-corrected *p* values for all pairwise correlations presented in Supplementary Table 1. Associations between the LDAEP slopes and STAI-T and HA scores are demonstrated in Figure 3. Significant correlations among the STAI and TCI items were observed, with strong correlations between NS and HA (*r* = −0.50), SD and HA (*r* = −0.52), C and RD (*r* = 0.60), STAI-T score and HA (*r* = 0.65), and STAI-T score and SD (*r* = −0.68) noted. Furthermore, sub-analysis results showed NS to be significantly correlated with the P50/N100 (*r* = 0.28) and N100/P200 (*r* = 0.36) slopes in partial correlation analysis controlled for sex (Table 3).

**Table 1:** LDAEP slopes.

**Stimulus (dB)**		**55**	**65**	**75**	**85**	**95**
Amplitude (μV)	P50/N100	6.6 (4.4)	7.7 (5.9)	8.3 (5.8)	9.5 (6.8)	11.0 (7.2)
	N100/P200	8.4 (5.2)	10.1 (6.2)	11.7 (6.6)	13.9 (7.9)	16.0 (7.9)
Latency (ms)	P50	57.7 (10.8)	52.5 (11.2)	51.8 (10.5)	51.3 (10.1)	48.3 (10.9)
	N100	102.0 (9.4)	99.3 (9.0)	98.2 (7.8)	96.9 (7.3)	97.3 (7.0)
	P200	204.8 (29.0)	202.8 (29.3)	194.4 (24.8)	192.9 (21.3)	195.6 (27.0)
Slope (μV/10dB)	P50/N100	1.0 (0.54)				
	N100/P200	1.8(0.95)				

Values are presented as the mean (SD).

SD, standard deviation.

**Table 2:** Influence of sex on each component.

	**Males (SD)**	**Females (SD)**	***P* value**
P50/N100 slope	0.08 (0.05)	0.11 (0.06)	0.08
N100/P200 slope	0.16 (0.09)	0.19 (0.09)	0.15
NS	12(3.4)	9.7 (3.5)	**0.0090** ^**^
HA	12 (4.7)	12 (4.7)	0.62
RD	9.3 (2.8)	9.7 (2.4)	0.58
P	2.1 (1.5)	2.7 (1.3)	0.11
SD	16 (4.4)	15 (5.6)	0.68
C	18 (2.7)	18 (3.4)	0.87
ST	3.1 (3.4)	4.3 (3.0)	0.13
STAI-S	39 (8.6)	41 (11)	0.39
STAI-T	44 (9.4)	47 (11)	0.24

^**^P < 0.01.

Sub-analysis of each variable was conducted using a t-test to determine sex differences.

SD, standard deviation; NS, novelty seeking; HA, harm avoidance; RD, reward dependence; P, persistence; SD, self-directedness; C, cooperativeness; ST, self-transcendence; STAI-S, State-Trait Anxiety Inventory-State Anxiety version; STAI-T, State-Trait Anxiety Inventory-Trait Anxiety version. Bold values visually emphasize statistically significant results.

**Table 3:** Partial correlation of NS with sex.

		**P50/N100**	**N100/P200**
NS	*r*	0.28	0.36
	*P*	0.030^*^	0.0050^**^

^*^P < 0.05.

^**^P < 0.01.

For variables with significant sex differences, partial correlation analyses were performed after adjusting for sex.

NS, novelty seeking.

## 4 Discussion

### 4.1 Associations between LDAEP and STAI-T

All of the present participants were classified as healthy adults, though it is worth noting that several exhibited a moderate level of anxiety. Based on commonly used clinical research guidelines and population norms, a score of ≥40 on the STAI-S or STAI-T is generally considered to indicate a moderate to higher anxiety level (Spielberger et al., 1970) and a considerable percentage scored above that threshold on both scales. The present findings suggest that even in non-clinical populations, there is meaningful variability in anxiety levels, which may influence psychophysiological measures such as LDAEP.

While evidence on the relationship between anxiety and LDAEP in healthy adults is limited, previous studies have reported findings in patients with GAD. A previous study reported that patients with GAD exhibited lower LDAEP as compared to healthy controls (Senkowski et al., 2003), though a subsequent study found no significant difference between patients and healthy subjects (Park et al., 2010). Park et al. (2011) in a later study then noted that this inconsistency could be attributed to the heterogeneous nature of GAD, similar to MDD, and further suggested that LDAEP may predict treatment response to SSRIs in patients with GAD. Specifically, patients with a high LDAEP showed better responses to SSRIs. In the present study of healthy adults, STAI-T results showed a negative correlation between LDAEP and trait anxiety. Although our sample consisted only of healthy participants, the observed association between high trait anxiety and LDAEP is consistent with the previous report (Senkowski et al., 2003). Moreover, in the present study, some participants showed moderate or higher levels of trait anxiety on the STAI-T despite being classified as healthy adults. Taken together, these findings suggest that individuals with elevated trait anxiety, even in the absence of psychiatric diagnosis, may display neurophysiological patterns similar to those linked with poorer long-term outcomes in disorders such as GAD (Chambers et al., 2004). While the value of LDAEP as a diagnostic marker for GAD remains uncertain, this supports the potential use of LDAEP as a non-invasive biomarker to predict treatment response in anxiety-related conditions.

Although dipole source analysis (DSA) is widely used for LDAEP measurement and provides greater anatomical specificity, it is less feasible for clinical applications. For the present study, a single-electrode method with only a Cz electrode was employed (Hagenmuller et al., 2011). That study noted that a single-electrode method may include frontal components, thus the results may differ from those obtained using DSA and require cautious interpretation. Nevertheless, findings of the previous comparative study conducted by Park et al. (2011) demonstrated that LDAEP measured at the Cz could predict SSRI treatment response in patients with GAD across multiple clinical measures, including the Hamilton Anxiety Rating Scale, Clinical Global Impression-Severity Scale (CGI-S), and Beck Anxiety Inventory. In contrast, LDAEP recorded at Fz and Pz was found to only be associated with CGI-S, thus indicating a potential advantage of Cz electrode recordings. Given that the present study was conducted to examine anxiety-related characteristics, use of Cz electrode recordings was considered to be a reasonable approach. However, direct comparison of amplitude, latency, and slope obtained here with DSA-derived source measures is not feasible.

### 4.2 Associations between LDAEP and TCI

It has been reported that LDAEP is positively correlated with sensation seeking (SS) (Carrillo-de-la-Peña, 1992), while SS itself was found to be negatively associated with harm avoidance (HA) (Cloninger, 1994) and also serotonergic sensitivity (Netter et al., 1996), thus LDAEP may be negatively correlated with HA. In line with this speculation, there was a significant negative correlation between LDAEP and HA noted in the present study. On the other hand, a previous study of healthy participants conducted by Juckel et al. (1995) did not find a negative correlation between LDAEP and HA. One possible explanation for this discrepancy could be methodological differences, particularly use of DSA in that previous study as compared to the single-electrode method in the present one. Another important methodological consideration is sample composition, as the previous study only examined male participants. Interestingly, in a recent study conducted by Andersson et al. (2025), LDAEP was found to be higher in healthy adult females than males and negatively correlated with age in females. These findings indicate that both sex and age should be considered when interpreting LDAE *P* values, and may explain, at least in part, the differences observed between the studies.

It is also important to highlight the clinical implications of this association. HA is a personality trait that has been associated with treatment-resistant MDD (Takahashi et al., 2013), non-remission of MDD (Balestri et al., 2019), and recurrence of MDD (Teraishi et al., 2015). Thus, the observed negative correlation between LDAEP and HA in the present study suggests potential clinical relevance, supporting the possibility that LDAEP may be useful for predicting treatment response in MDD.

Beyond the relationship with HA, LDAEP was also linked to other TCI dimensions. Notably, NS exhibited a clear sex difference in our sample, with males scoring higher than females, which aligns with prior literature. Imaging studies have demonstrated sex differences in striatal dopamine release, supporting a neurobiological basis for higher NS in males (Cloninger, 1994; Munro et al., 2006). From a psychosocial perspective, prior research has shown that males consistently score higher on SS and risk-taking than females (Cross et al., 2011). Taken together, these biological and psychosocial influences provide a plausible explanation for the observed sex difference in NS. Given this sex difference, partial correlation analysis controlling for sex was conducted, and the results indicated a positive correlation between LDAEP and NS, consistent with Juckel et al. (1995). In contrast, no significant correlation was observed when all participants were analyzed together. Although direct confirmation was not obtained, the present findings are broadly consistent with previous research and emphasize the importance of considering sex differences (Oliva et al., 2011; Jaworska et al., 2012; Fujita et al., 2022).

For analyzing the association between LDAEP and NS, it is important to consider neurotransmitter systems beyond serotonin. Previous studies have found NS to be positively correlated with plasma norepinephrine level (Gerra et al., 1999) and also associated with the genotype of the norepinephrine transporter (Lee et al., 2008). Additionally, others have provided findings linking NS to the genotype of the D4 dopamine receptor gene (Ebstein et al., 1996; Benjamin et al., 1996), suggesting involvement of the dopaminergic system. On the other hand, LDAEP has been shown to predict treatment response to reboxetine, a selective noradrenergic agent, in a pattern opposite to that observed with SSRIs (Juckel et al., 2007; Cerda and Fitzgerald, 2021). Furthermore, a genotype known to be associated with reduced catechol-O-methyltransferase activity, which indicates altered dopaminergic neurotransmission, has been found to be linked to lower LDAE *P* values (Juckel et al., 2008), while LDAEP has also been reported to have a positive correlation with dopamine transporter availability, in contrast to its negative correlation with serotonin transporter availability (Lee et al., 2011). Thus, even though LDAEP is commonly regarded as a marker of central serotonergic function, evidence indicating its relevance to noradrenergic and dopaminergic systems is emerging (Fitzgerald, 2024). The present study found LDAEP to be positively correlated with NS, providing additional support for the growing perspective that LDAEP may reflect not only serotonergic activity, but also broader monoaminergic influences, including those of noradrenaline and dopamine.

Cronbach's alpha coefficients for the seven TCI dimensions in the present study were as follows: NS = 0.65, HA = 0.84, RD = 0.61, *P* = 0.57, SD = 0.82, C = 0.64, and ST = 0.80, which indicate acceptable to good internal consistency for most, particularly HA, SD, and ST. The reliability of the *P* and RD scales was relatively low, though consistent with previous reports indicating that scales with fewer items or multidimensional constructs tend to yield lower alpha values. Notably, these coefficients are comparable to those reported by Kijima (1996), whose findings were used to develop and validate the 125-item Japanese version of the TCI. They reported alpha coefficients in a similar range (e.g., HA = 0.86, SD = 0.82, *P* = 0.57), thus providing support for the internal consistency of the Japanese TCI even in its dichotomous (yes/no) response format. While the present findings indicate that this instrument demonstrates acceptable reliability, it may be helpful for future studies to consider use of a four-point Likert scale, which has been shown to improve internal consistency and measurement precision when using the Japanese version of the TCI.

### 4.3 Trait interactions and selective associations with LDAEP

HA showed a strong negative correlation with NS and SD, and a moderate to strong positive correlation with STAI-S and STAI-T. Furthermore, STAI-T demonstrated a weak negative correlation with NS and a strong negative correlation with SD, as well as a moderate positive correlation with STAI-S. RD was strongly and positively correlated with C. These results are consistent with those of a previous study of a cohort of medical students and medical staff that found anxiety, assessed using the Temperament Evaluation of Memphis, Pisa, Paris, and San Diego Auto-questionnaire, to be negatively correlated with NS and SD, and positively correlated with HA, measured using the TCI (Shirahama et al., 2018).

Although LDAEP was shown to be correlated with NS, HA, and STAI-T scores, no significant relationship was observed with other psychological measures correlated with these traits. These results raise the possibility that LDAEP and the specific psychological traits NS, HA, and STAI-T may share common underlying pathophysiological processes—for example, modulation of cortical excitability by serotonergic as well as dopaminergic and noradrenergic neurotransmitter systems. Moreover, tonic serotonergic and noradrenergic control may exert opposing effects on LDAEP, which should be taken into account in the interpretation of psychological data (Fitzgerald, 2024), though further research is needed for confirmation. Notably, no such associations were found with other personality dimensions, suggesting a degree of specificity observed in the observed relationships. The present exploratory findings are considered to provide insight into the neurophysiological correlates of LDAEP, as well as its relationships with certain temperament and anxiety-related traits. In contrast, LDAEP may not be suitable for reflecting state anxiety, though the cross-sectional and correlational nature of the present study precludes causal interpretation or definitive conclusions regarding LDAEP as a trait biomarker. Therefore, these associations must be interpreted with caution. Longitudinal and interventional studies will be necessary to clarify whether LDAEP can serve as a reliable marker of enduring personality traits and/or anxiety vulnerability.

Studies regarding use of the LDAEP for healthy adults are limited, which makes it difficult to clearly interpret the present findings or fully understand their physiological significance. Considering the need for practical and reproducible methodologies in clinical settings, the present study employed what is arguably the simplest available approach. Despite its methodological simplicity, the LDAEP approach may have meaningful utility as a non-invasive, cost-effective indicator of central neurotransmitter activity, including serotonergic, dopaminergic, and noradrenergic systems. With further validation, it could serve as a screening tool in both research settings and potentially in clinical practice to identify individuals with altered neurotransmission profiles.

## 5 Limitations

It is essential to perform clinical and experimental research using appropriate methods. Nevertheless, the present study has several limitations that should be considered. First, while several significant correlations were observed, multiple comparison correction was not applied due to its hypothesis generation nature. Therefore, the findings should be interpreted with caution and future studies will be needed for confirmation. Second, the number of participants was relatively small, thus future related investigations will need a greater number of participants to validate the present results. Third, some participants exhibited low-amplitude deflections that resulted in unclear peaks. However, to obtain findings in line with previous studies, the measurements were performed based on measurements of P50 components with a maximum amplitude of 30–80 ms. Fourth, each participant noted in self-reporting that they had no hearing impairment and an actual hearing test was not performed. Finally, intra- and inter-subject variability of LDAEP parameters (amplitude and latency) was not assessed in the present study, which may have influenced the findings through unmeasured within- and between-participant fluctuations. Future studies should incorporate test–retest designs to evaluate the stability and reproducibility of LDAEP parameters.

## 6 Conclusion

This study provides novel evidence suggesting an inverse relationship between LDAEP, trait anxiety, and HA in healthy adults. In addition, in analysis controlled for sex, NS was found to be significantly correlated with LDAEP. These findings are anticipated to be useful for future research related to the neurophysiological basis of certain temperament and anxiety-related traits.

## Data Availability

The raw data supporting the conclusions of this article will be made available by the authors, without undue reservation.
