# Supplementary material for: Loudness dependence of auditory evoked potentials reflects trait anxiety and harm avoidance in healthy adults: an exploratory study
Source: Front Hum Neurosci. 2025 Oct 15;19:1615407. doi: 10.3389/fnhum.2025.1615407 (PMC12568648; doi:10.3389/fnhum.2025.1615407)
Supplement: Supplementary file 1 [file Data_Sheet_1.pdf]

Supplementary Material

Supplementary Figure 1

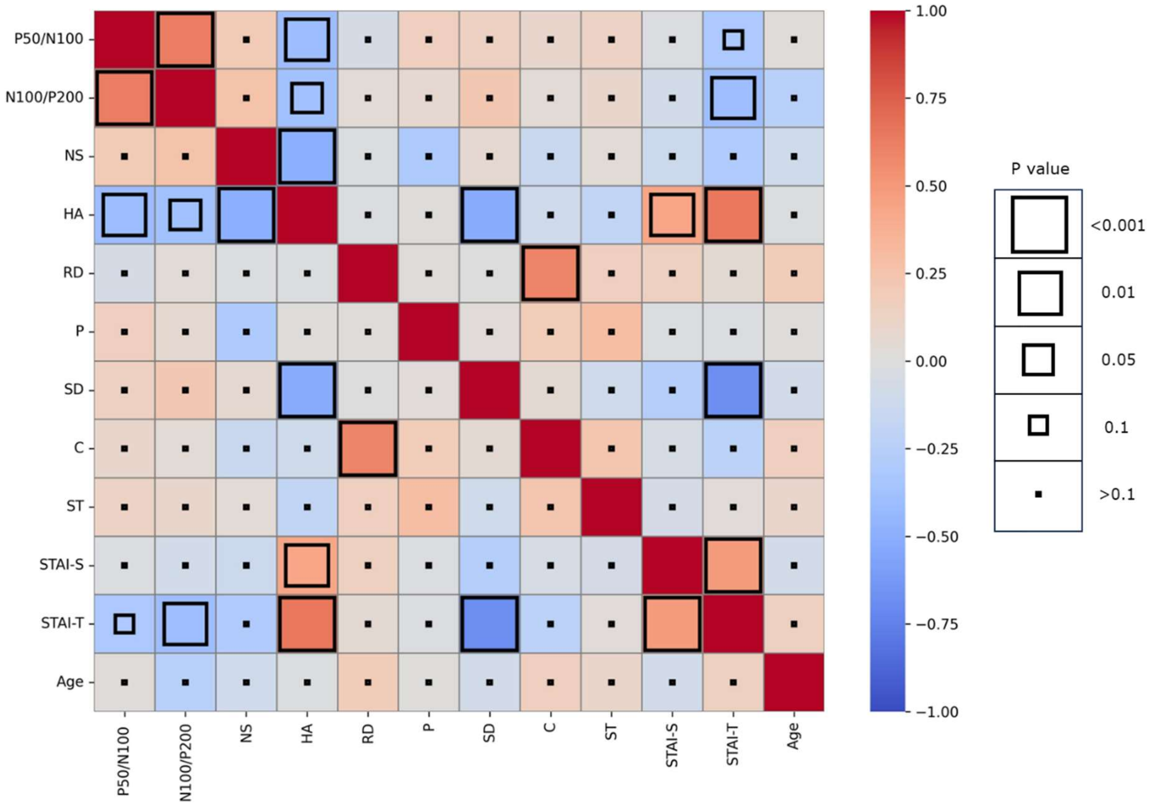

Supplementary Figure 1. Heatmap showing pairwise Spearman correlation coefficients between LDAEP, psychological measures, and age, with FDR-corrected p-values (Benjamini–Hochberg).

Color intensity indicates the strength and direction of the correlation, while the size of each square represents the statistical significance based on FDR-corrected p-values using the Benjamini–Hochberg procedure.

Supplementary Table 1. Pairwise Spearman correlation coefficients (r) between LDAEP and psychological measures, with raw and FDR-corrected (Benjamini–Hochberg) p-values.

Variable 1	Variable 2	r	Raw P-value	Adjusted P-value (FDR, BH method)
P50/N100	N100/P200	0.63	0.000000070**	0.0000015**
P50/N100	NS	0.20	0.14	0.42
P50/N100	HA	-0.41	0.0012**	0.0088**
P50/N100	RD	-0.06	0.64	0.89
P50/N100	P	0.16	0.21	0.53
P50/N100	SD	0.14	0.27	0.57
P50/N100	C	0.09	0.51	0.84
P50/N100	ST	0.13	0.34	0.68
P50/N100	STAI-S	-0.02	0.90	1.0
P50/N100	STAI-T	-0.32	0.012*	0.066
P50/N100	Age	0.02	0.90	1.0
N100/P200	NS	0.26	0.048*	0.19
N100/P200	HA	-0.38	0.0026**	0.016*
N100/P200	RD	0.04	0.76	0.96
N100/P200	P	0.07	0.61	0.89
N100/P200	SD	0.22	0.090	0.30

N100/P200	C	0.04	0.74	0.96
N100/P200	ST	0.09	0.49	0.83
N100/P200	STAI-S	-0.08	0.54	0.87
N100/P200	STAI-T	-0.40	0.0014**	0.0092**
N100/P200	Age	-0.25	0.054	0.20
NS	HA	-0.50	0.000056**	0.00062**
NS	RD	-0.01	0.92	1.0
NS	P	-0.30	0.020*	0.10
NS	SD	0.07	0.57	0.87
NS	C	-0.14	0.28	0.58
NS	ST	0.04	0.74	0.96
NS	STAI-S	-0.12	0.37	0.72
NS	STAI-T	-0.29	0.030*	0.13
NS	Age	-0.10	0.43	0.81
HA	RD	-0.01	0.97	1.0
HA	P	0.01	0.94	1.0
HA	SD	-0.52	0.000020**	0.00026**
HA	C	-0.10	0.44	0.81
HA	ST	-0.18	0.16	0.44
HA	STAI-S	0.43	0.00063**	0.0052**

HA	STAI-T	0.65	0.000000027**	0.00000089**
HA	Age	-0.01	0.95	1.0
RD	P	0.02	0.91	1.0
RD	SD	0.00	0.99	1.0
RD	C	0.60	0.00000041**	0.0000068**
RD	ST	0.16	0.23	0.54
RD	STAI-S	0.15	0.25	0.57
RD	STAI-T	0.06	0.64	0.89
RD	Age	0.19	0.15	0.43
P	SD	0.03	0.82	1.0
P	C	0.18	0.16	0.44
P	ST	0.29	0.02*	0.10
P	STAI-S	-0.01	0.95	1.0
P	STAI-T	-0.02	0.88	1.0
P	Age	0.01	0.93	1.0
SD	C	0.06	0.63	0.89
SD	ST	-0.10	0.46	0.82
SD	STAI-S	-0.26	0.046*	0.19
SD	STAI-T	-0.68	0.0000000027**	0.00000018**
SD	Age	-0.08	0.56	0.87

C	ST	0.24	0.06	0.21
C	STAI-S	-0.05	0.72	0.95
C	STAI-T	-0.22	0.10	0.31
C	Age	0.16	0.22	0.54
ST	STAI-S	-0.07	0.61	0.89
ST	STAI-T	0.03	0.84	1.0
ST	Age	0.10	0.47	0.82
STAI-S	STAI-T	0.49	0.000076**	0.00072**
STAI-S	Age	-0.08	0.55	0.87
STAI-T	Age	0.15	0.26	0.57

* p-value < 0.05.

** p-value < 0.01.
